# Immunomodulatory Properties of Multi-Strain Postbiotics on Human CD14^+^ Monocytes

**DOI:** 10.3390/life14121673

**Published:** 2024-12-17

**Authors:** Kyle D. Roberts, Sadia Ahmed, Erin San Valentin, Luca Di Martino, Thomas S. McCormick, Mahmoud A. Ghannoum

**Affiliations:** 1 Department of Dermatology, Case Western Reserve University, Cleveland, OH 44106, USA; 2 Case Digestive Health Research Institute, School of Medicine, Case Western Reserve University, Cleveland, OH 44106, USA; 3 Department of Dermatology, University Hospitals Cleveland Medical Center, Cleveland, OH 44106, USA

**Keywords:** *Lactobacillus acidophilus*, *Lacticaseibacillus rhamnosus*, *Saccharomyces boulardii*, immunomodulation, postbiotics

## Abstract

The ability of probiotics, comprising live microbiota, to modulate the composition of intestinal microbiomes has been connected to modulation of the central nervous system (Gut–Brain axis), neuroendocrine system (Gut–Skin axis), and immune response (Gut–Immune axis). Less information is known regarding the ability of postbiotics (cell wall components and secreted metabolites derived from live organisms) to regulate host immunity. In the present study, we tested postbiotics comprising single strains of bacteria and yeast (*Lactobacillus acidophilus* 16axg, *Lacticaseibacillus rhamnosus* 18fx, *Saccharomyces cerevisiae var. boulardii* 16mxg) as well as combinations of multiple strains for their ability to stimulate cytokine production by human CD14^+^ monocytes. We quantified cytokine gene and protein expression levels in monocytes following stimulation with postbiotics. Both heat-killed *L. acidophilus* and *L. rhamnosus* stimulated naïve monocytes without significant differences between them. Heat-killed *S. boulardii* stimulated less cytokine production compared to postbiotic bacteria at the same concentration. Interestingly, the addition of heat-killed yeast to heat-killed *L. acidophilus* and *L. rhamnosus* resulted in an enhancement of immune stimulation. Thus, heat-killed postbiotics have immune-modulating potential, particularly when bacteria and yeast are combined. This approach may hold promise for developing targeted interventions that can be fine-tuned to modulate host immune response with beneficial health impact.

## 1. Introduction

Improvement of human health through the modulation of microbial interactions is surging and increasingly becoming more significant for consumers, manufacturers, healthcare professionals, and regulators. In this context, probiotics, which are live microorganisms that confer health benefits when administered adequately, play a crucial role [1]. Probiotic supplementation has the potential to alleviate digestive discomfort [2], improve electrolyte absorption [3], and facilitate essential amino acid and vitamin absorption in the intestine [4,5]. However, fully realizing this potential remains difficult, in part due to the unavoidable loss in viability as probiotics traverse through the challenging stomach environment. These challenges have prompted exploring alternative preparations of probiotics which retain their health benefits but offer more stability. These preparations are termed “postbiotics”, which are defined as “preparations of inanimate microorganisms and/or their components that confer a health benefit on the host” [6]. Compared to probiotics, postbiotics are more stable over a wider range of environmental conditions such as temperature and pH, and thus provide practical advantages from a production standpoint [7].

Probiotics have been postulated to regulate host immunity by influencing the immune response of both the innate and adaptive arms of the immune system [8,9]. Together, these two arms work to maintain immunological tolerance of commensal bacteria and dietary antigens while simultaneously identifying and combating opportunistic pathogens that may cause infections [10,11,12]. Innate immunity is promoted via the recognition of microbe-associated molecular patterns (MAMPs) by pattern recognition receptors (PRRs) such as Toll-like receptors (TLRs) found in myeloid cell lineages, including neutrophils, macrophages, and dendritic cells [13]. Systemic adaptive immune responses beyond the gut can be influenced by the gut microbiota through immune cell priming of lymphocytes, which traverse throughout the mucosa-associated lymphoid tissue (MALT) [10].

Probiotics and postbiotics have been proposed to support an immunomodulatory role in the gut microbiome [14]. Moreover, similar to live probiotics, postbiotics can induce protective modulation against pathogenic organisms through direct antimicrobial properties (i.e., bacteriocins) and competition for adhesion sites (i.e., fimbriae), fortifying the epithelial barrier, and modulating immune responses [6,15]. A recent example of postbiotic modulation has been illustrated by heat-killed *Lactobacillus acidophilus* that exhibited strong adhesive properties with undifferentiated cells, enterocytic cells, and the mucus layer [16]. Additionally, Coconnier et al. [17] used heat-killed *L. acidophilus* to demonstrate a dose-dependent inhibition of cell association and invasion by different diarrhea-inducing bacteria, including *Escherichia coli*, *Listeria monocytogenes*, *Yersinia pseudotuberculosis*, and *Salmonella typhimurium*. Another study investigated the anti-inflammatory potential of bacterial cell wall components of heat-killed *Lacticaseibacillus rhamnosus GG* on a co-culture model of intestinal inflammation, demonstrating that the cell wall components were capable of reducing LPS-induced inflammation [18].

Recently, postbiotic yeast preparations, particularly *Saccharomyces (S) cerevisiae var. boulardii*, have been gaining interest for their health benefits. An in vivo murine study using a dextran sodium sulfate (DSS)-induced colitis model demonstrated that both freeze-dried and spray-dried postbiotic *S. boulardii* reduced the secretion of proinflammatory factors and increased the secretion of anti-inflammatory factors [19]. In addition, the gut microbiome was found to be regulated, showing effective restoration of dominant beneficial bacterial phyla such as *Bacteroidota* and *Actinobacteriota* in the DSS-induced colitis mice supplemented with the yeast postbiotic [19].

While single-strain postbiotic treatments have been explored, our study focuses on elucidating the immunomodulatory properties of multi-strain postbiotic formulations comprising heat-killed beneficial bacteria [*Lactobacillus acidophilus 16axg* (LA), *Lacticaseibacillus rhamnosus 18fx* (LR)] combined with the fungus *Saccharomyces cerevisiae var. boulardii 16mxg* (SB). We compared the potential combinatorial effects of the postbiotics on the activation of CD14^+^ human peripheral blood monocytes (PBMCs). Additionally, we explored various ratios of the combination postbiotic mixture compared to single organisms, as well as a time course of immune induction including cytokine activity elicited by activation with multiple postbiotic formulations. Our results demonstrate that both heat-killed *L. acidophilus* and *L. rhamnosus* stimulate naïve monocytes without significant differences between them. Although heat-killed *S. boulardii* was less immunomodulatory, the addition of heat-killed yeast to heat-killed bacteria resulted in enhanced immune stimulation.

## 2. Materials and Methods

### 2.1. Postbiotic Preparation

*L. acidophilus* and *L. rhamnosus* were heat-killed at 70 °C for 30 min, lyophilized, and supplied (BioSource Culture and Flavors, Muskego, WI, USA) at a concentration of 1 × 10^11^ colony-forming units (CFUs)/g. The heat-killing of the bacteria was verified by plating on Mann, Rogosa, Sharpe Agar (MRSA) (Sigma Aldrich, St. Louis, MO, USA) and incubating at 37 °C for 24 h in a Bactron 300 anaerobic chamber (Sheldon Labs, Cornelius, OR, USA). Lyophilized powder was weighed and resuspended in RPMI 1640 (Gibco, Grand Island, NY, USA) at a concentration of 10 mg/mL. *S. boulardii* was cultured in Sabouraud Dextrose Broth (BD, Sparks, MD, USA) with 5% Dextrose for 24 h at 37 °C. The *S. boulardii* cultures were centrifuged at 2500× *g*, washed in phosphate-buffered saline (PBS), centrifuged, and resuspended in PBS. The resuspended pellets were incubated in a Model 10 Lab benchtop oven (Quincy Lab Inc., Burr Ridge, Chicago, IL, USA) at 70 °C for 30 min to heat-inactivate the yeast. The heat-killing of the yeast was verified by plating on Potato Dextrose Agar (PDA) and incubating at 37 °C for 24 h. The pellets were centrifuged at 3000× *g*, and the supernatant was discarded. The wet mass of the pellet was quantified. The pellets were then resuspended in RPMI at a concentration of 10 mg/mL (mg heat-killed organism (s)/mL). *S. boulardii* was combined with heat-killed *L. acidophilus* and *L. rhamnosus* for a final concentration of 10 mg/mL of all three organisms at a 1:1:1 ratio (3.33 mg/mL each).

### 2.2. Primary PBMC Sourcing and Culture

Human volunteer studies were approved by the Institutional Review Board of University Hospitals Cleveland Medical Center (UHCMC, IRB# 05-95-03). All procedures were performed in accordance with Declaration of Helsinki principles. Six healthy blood donors, four males and two females all between the ages of 24 and 57 years old, were recruited to donate 120 mL of whole blood at the Department of Dermatology University Hospitals Cleveland Medical Center Skin Diseases Research Center with informed consent. Peripheral blood was obtained using vacutainers containing 0.01% (*w*/*v*) EDTA (BD, Sparks, MD, USA). All PBMC cell culture was performed using RPMI 1640 without glutamine (Gibco, Grand Island, NY, USA), supplemented with 10% (*v*/*v*) Fetal Bovine Serum (Gibco, Grand Island, NY, USA) and 1% (*v*/*v*) Penicillin–Streptomycin (Gibco, Grand Island, NY, USA). Cells were cultured in untreated sterile cultureware except where specifically indicated otherwise. Monocytes from donors 1–5 were utilized for both RT-qPCR and Luminex analysis, while monocytes from donor 6 were only utilized for Luminex analysis.

### 2.3. CD14^+^ Monocyte Isolation

CD14^+^ cells were isolated from whole-blood donations as described previously [20]. Whole blood was collected in vacutainers with EDTA and immediately processed for PBMC isolation. Whole blood was diluted with prewarmed Hanks’s buffered saline solution (HBSS) without divalent cations, followed by layering onto Histopaque^®^ 1077 (Sigma Aldrich, St. Louis, MO, USA) and centrifuging for 20 min without braking. Buffy coats containing leukocytes were collected, and red blood cells were lysed using ACK lysis buffer (Gibco, Grand Island, NY, USA) at room temperature for 5 min. After centrifugation and cell counting, PBMCs were resuspended and incubated with anti-CD14 microbeads (Miltenyi Biotec, Auburn, CA, USA) for 15 min at 4 °C with gentle agitation. Following this, cells were centrifuged, washed, and resuspended in PBS. The resuspended cells were passed through an LS column (Miltenyi Biotec, Auburn, CA, USA) in a MACS^®^ separator (Miltenyi Biotec, Auburn, CA, USA), which was then washed three times with PBS. The column was then removed from the MACS^®^older, and the bound cells were eluted with fresh media. CD14^+^ monocytes were counted and resuspended to 1 × 10^6^ cells/mL for subsequent experiments.

### 2.4. Exposure of CD14^+^ Monocytes to Postbiotics

CD14^+^ monocytes were incubated in 0.8 mL V-bottomed 96-well plates in RPMI 1640 without glutamine with 10% FBS and 1% PE/ST. Monocytes were challenged with prepared postbiotics at a final concentration of 100 µg/mL and incubated at 37 °C in a humidified atmosphere with 5% CO_2_. After 2 h, 4 h, 8 h, and 24 h, the monocytes were centrifuged at 250× *g* and the supernatant was collected and stored at −80 °C, while cell pellets were retained for total RNA extraction.

### 2.5. RNA Extraction

CD14^+^ cell pellets were processed for RNA extraction using the RNeasy 96 extraction kit (Qiagen, Hilden, Germany) on the QIACube HT (Qiagen, Hilden, Germany) according to the manufacturer’s instructions [21]. RNA quantity and quality were verified using a broad-range RNA assay on the Qubit 2.0 (Invitrogen, Waltham, MA, USA) and a Nanodrop One (Thermo Scientific, Waltham, MA, USA) for 5 random samples for each run.

### 2.6. TaqMan Probe Design

TaqMan probes were designed for four human gene targets: glyceraldehyde 3-phosphate dehydrogenase (*GAPDH*), interleukin 6 (*IL-6*), interleukin 10 (*IL-10*), and tumor necrosis factor alpha (*TNFα*) [22]. Each probe was ordered with a different fluorescent dye to allow for multiplexing (Integrated DNA Technologies, Inc (IDT), Coralville, IA, USA). Primers were designed using PrimerQuest (IDT, Coralville, IA, USA). Primer and probe sequences, dyes, and quenchers are listed in the Supplementary Materials (Table S1).

### 2.7. RT-qPCR

Reverse transcription–quantitative polymerase chain reaction (RT-qPCR) was performed using two technical replicates using TaqPath 1-Step Multiplex Master Mix (No ROX) (Invitrogen, Waltham, MA, USA) according to the product guide. Two Master Mixes were created to allow for multiplexing: one mix contained probes and primers for *IL-6* and *IL-10*, and the other mix contained primer and probe sets for *GAPDH* and *TNFα*. The thermal cycling conditions are listed in the Supplementary Materials (Table S2). RT-qPCR was performed on a qTower3 (Analytik, Jena, Upland, CA, USA). Relative fold change was determined using the ΔΔCT method normalized to *GAPDH* expression levels [20].

### 2.8. Luminex Analysis

Supernatants harvested from 24 h CD14^+^ PBMC cultures (*n* = 6) were aliquoted and stored at −80 °C prior to analysis. Supernatants were thawed on wet ice and cytokine abundance was measured using the ProcartaPlex^TM^ 45 Plex Cytokine and Growth Factor Assay (Invitrogen, Waltham, MA, USA). Quantification was performed using a Luminex^®^ 100/200™ System (Luminex Corporation, Austin, TX, USA) according to the manufacturer’s protocol [23]. Each sample was measured in duplicate wells. Aliquots of sterile media were used as blanks. A five-parameter logistic (5 PL) curve was fit to process the multiplex data.

### 2.9. Statistical Analysis

Statistical analysis was performed in R (version 4.3.2) and RStudio (2024.04.1 + 748 “Chocolate Cosmos”) using a one-way repeated-measures ANOVA with an applied Greenhouse–Geisser correction for within-subject factors that violated the sphericity assumption (Mauchly’s test, *p* ≤ 0.05). Post hoc comparisons between samples were made using paired *t*-tests with Holm’s correction for multiple comparisons. For post hoc comparisons, an adjusted *p*-value of *p.*adj < 0.05 was considered significant.

## 3. Results

### 3.1. Immune Response Timing

**Figure 1:**
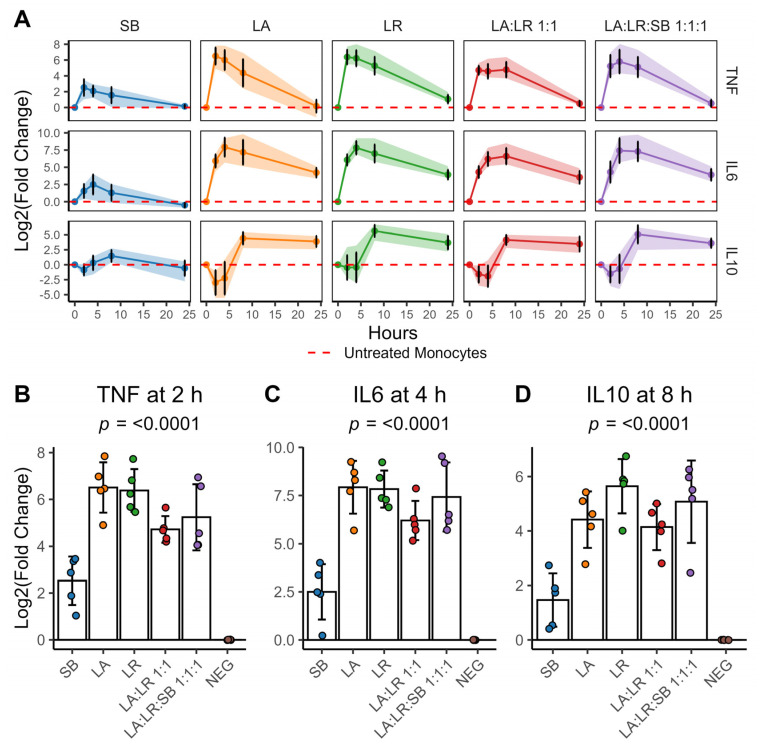
CD14^+^ monocytes have increased expression of proinflammatory genes following postbiotic stimulation, followed by increased gene expression of anti-inflammatory signals. (**A**). Time course of *TNFα*, *IL-6*, and *IL-10* expression in CD14^+^ monocytes up to 24 h after exposure to heat-killed postbiotics. Points indicate the sample mean for each timepoint; error bars represent the mean ± standard deviation. The highlighted ribbon indicates the range between the maximum and minimum values observed at each timepoint over 24 h. The red dashed line indicates the normalized level of gene expression in the untreated control. (**B**). *TNFα* gene expression in CD14^+^ monocytes cultured with heat-killed postbiotics after 2 h. (**C**). *IL-6* gene expression in CD14^+^ monocytes cultured with heat-killed postbiotics after 4 h. (**D**). *IL-10* gene expression in CD14^+^ monocytes cultured with heat-killed postbiotics after 8 h. For (**B**–**D**), error bars represent the sample mean ± standard deviation. Colors indicate the treatment group, while individual data points represent individual donors (*n* = 5 per treatment). The sample means were compared using a repeated-measures ANOVA with an applied Greenhouse–Geisser correction for within-subject factors. An adjusted *p*-value of *p* < 0.05 was considered significant.

Heat-killed postbiotics were prepared comprising each organism individually (LA, LR, SB), equal amounts of both lactic acid bacteria (LA:LR 1:1), and equal amounts of both lactic acid bacteria and yeast (LA:LR:SB 1:1:1). In order to initially characterize the potential inflammatory activity of the postbiotics, *TNFα* and *IL-6* were selected as proinflammatory markers, while *IL-10* was chosen as a marker for anti-inflammatory activity. Cytokine gene expression was time- and dose-dependent, with different peak expression times for each target gene (Figure 1A). *TNFα* gene expression in CD14^+^ monocytes peaked 2 h after stimulation with heat-killed bacterial and yeast cells then declined after 8 h (Figure 1A). In contrast, *IL-6* and *IL-10* gene expression peaked at 4 and 8 h, respectively, and remained elevated compared to untreated controls for up to 24 h post-stimulation (Figure 1A). Interestingly, *IL-10* gene expression was downregulated relative to untreated controls (NEG) initially following stimulation. This suggests that heat-killed bacterial and yeast cells may initially activate CD14^+^ monocytes towards a proinflammatory response via two separate but related mechanisms. In the first 4 h post-stimulation, there was a simultaneous increase in proinflammatory (*IL-6* and *TNFα*) cytokine gene expression and a decrease in anti-inflammatory (*IL-10*) gene expression.

At all timepoints, CD14^+^ monocytes co-cultured with heat-killed *S. boulardii* had the lowest levels of gene expression for all targets. Treatment with a 1:1 combination of heat-killed *L. acidophilus* and *L. rhamnosus* significantly reduced peak *TNFα* expression levels in CD14^+^ monocytes compared to treatment with either species individually (Figure 1B, Table S3). Each of the postbiotic combinations containing heat-killed bacterial cells induced a significant increase in *IL-6* gene expression compared to CD14^+^ monocytes stimulated with yeast alone (Figure 1C, Table S4). The postbiotic combination consisting of both *L. acidophilus* and *L. rhamnosus* (LA:LR 1:1) significantly decreased peak *IL-10* gene expression in CD14^+^ monocytes compared to single-species *L. rhamnosus* postbiotics (Figure 1D, Table S5). Notably, the further addition of *S. boulardii* to the two bacterial species reversed this trend.

### 3.2. Characterization of Cytokines Following Co-Culture with Postbiotics

**Figure 2:**
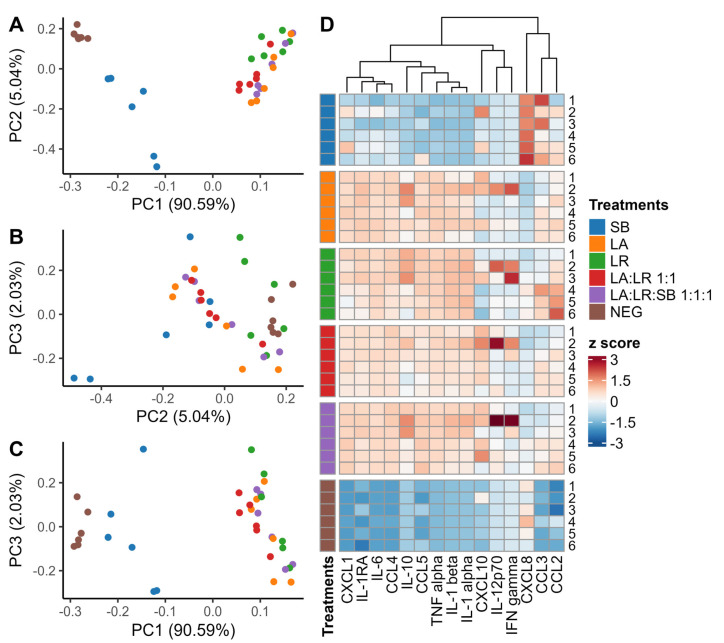
Differential cytokine production from CD14^+^ monocytes cultured with heat-killed postbiotics after 24 h. (**A**–**C**) Principal component analysis of supernatants of CD14^+^ monocytes 24 h after stimulation with heat-killed postbiotics. Colors indicate the treatment group, while individual data points represent individual donors (*n* = 6 per treatment). (**D**) Heatmap with a complete linkage hierarchical clustering for scaled cytokines of interest. Each break in the heatmap represents a different treatment, and each row is labeled by donor number.

Protein levels of 45 cytokines and growth factors secreted by treated and untreated (NEG) CD14^+^ monocytes after 24 h co-culture with postbiotics were quantified using Luminex xMAP technology (Luminex Corporation, Austin, TX, USA) (Figure S1). Low-abundance cytokines, defined as cytokines without a single sample with a Mean Fluorescence Intensity (MFI) > 4000, were excluded from further analysis. Principal component analysis (PCA) was performed using all cytokines above the intensity cutoff (Figure 2A–C). Complete linkage hierarchical clustering of cytokines above the intensity cutoff was performed using the Euclidean distance of the scaled MFI values for each cytokine (Figure 2D).

As a precaution, the relative levels of cytokine and growth factor concentrations in undiluted individual donor samples were compared to the concentrations in diluted pooled donor samples to assess possible postzoning in 15 target cytokines above the MFI cutoff. Postzoning is a false negative phenomenon that can occur in sandwich immunoassays. Excess antigen may saturate both the capture and detection antibodies separately, preventing the formation of an antibody “sandwich”, thus causing an artificially low fluorescence intensity for highly abundant antigens. The following cytokines showed an inconsistent fluorescence pattern in undiluted individual donor samples compared to diluted samples of pooled supernatant from each donor: CC chemokine ligand 3 (*CCL3*), CXC chemokine ligand 8 (*CXCL8*), and CC chemokine ligand 2 (*CCL2*) (Figure S2A–F). This observation suggested that the reduced fluorescence intensity observed for these cytokines in undiluted samples may be due to postzoning. All samples that were observed to have evidence of postzoning were excluded from further analysis. Additionally, CXC chemokine ligand 10 (*CXCL10*), interleukin 12 subunit 70 (*IL-12p70*), and interferon gamma (*IFNγ*) were in low abundance for the majority of samples, with only monocytes from a single donor producing a large amount of cytokine (Figure S3A–C). Cytokines exhibiting this pattern were also excluded from further analysis.

### 3.3. Chemokine Production

**Figure 3:**
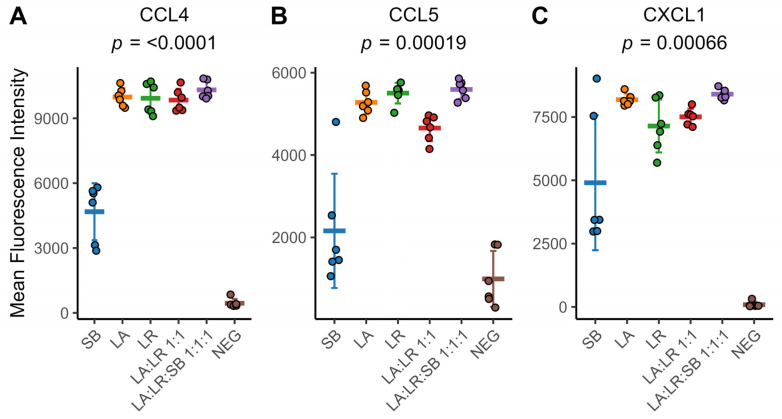
The inclusion of *S. boulardii* in combination with *Lactobacillus sp*. increases chemokine abundance in the supernatant of primary CD14^+^ monocytes following 24 h of co-culture with heat-killed postbiotics. (**A**) *CCL4* fluorescent intensity after 24 h culture of CD14^+^ monocytes stimulated with heat-killed bacteria. (**B**) *CCL5* fluorescent intensity after 24 h culture of CD14^+^ monocytes cultured with heat-killed bacteria. (**C**) *CXCL1* fluorescent intensity after 24 h co-culture of CD14^+^ monocytes with heat-killed bacteria. For (**A**–**C**), individual colored points represent individual donors (*n* = 6), the thick colored line indicates the sample mean for all donors, and error bars represent the sample mean ± standard deviation. The sample means were compared using a repeated-measures ANOVA with an applied Greenhouse–Geisser correction for within-subject factors. An adjusted *p*-value of *p* < 0.05 was considered significant.

CD14^+^ monocytes produced high levels of three chemokines in response to all postbiotic treatments (Figure 3A–C). The postbiotic combination of heat-killed bacteria and yeast (LA:LR:SB 1:1:1) created an additive effect that significantly increased the production of these chemokines compared to heat-killed bacteria without yeast (LA:LR 1:1). The postbiotic combination that on average induced the greatest production of CC chemokine ligand 4 (*CCL4*), CC chemokine ligand 5 (*CCL5*), and CXC chemokine ligand (*CXCL1*) was LA:LR:SB 1:1:1.

Monocytes from individual donors stimulated with heat-killed yeast only (SB) exhibited considerable inter-personal variability in chemokine production compared to monocytes stimulated with heat-killed bacteria. Monocytes stimulated with both heat-killed bacteria and yeast (LA:LR:SB 1:1:1) produced significantly more *CCL4*, *CCL5*, and *CXCL1* compared to monocytes stimulated with both heat-killed bacteria without yeast (LA:LR 1:1) (Figure 3A–C, Tables S6–S8). These results suggest a possible combinatorial effect, whereby the addition of heat-killed yeast to heat-killed bacteria induces greater cytokine production than heat-killed bacteria alone. Interestingly, monocytes stimulated with a postbiotic comprising both *L. acidophilus* and *L. rhamnosus* (LA:LR 1:1) produced significantly less *CCL5* compared to monocytes stimulated with either bacterial species individually (LA and LR) (Table S7).

### 3.4. Cytokine Production

**Figure 4:**
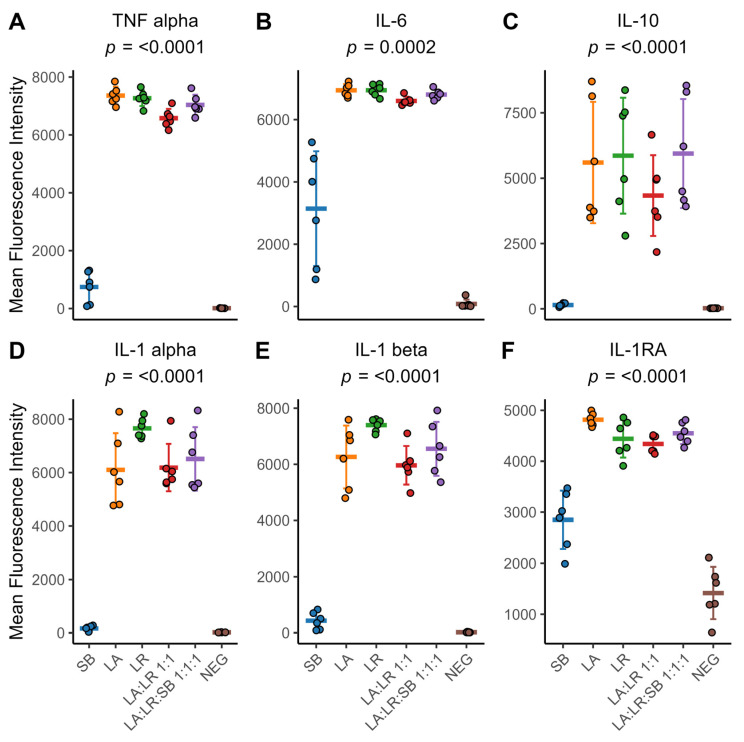
Cytokine production by primary CD14^+^ monocytes after 24 h of co-culture with heat-killed postbiotics. (**A**) *TNFα* fluorescence intensity. (**B**) *IL-6* fluorescence intensity. (**C**) *IL-10* fluorescence intensity. (**D**) *IL-1α* fluorescence intensity. (**E**) *IL-1β* fluorescence intensity. (**F**) IL-1 receptor antagonist fluorescence intensity. For (**A**–**F**), individual colored points represent individual donors, the thick colored line indicates the sample mean for all donors (*n* = 6), and error bars represent the sample mean ± standard deviation. The sample means were compared using a repeated-measures ANOVA with an applied Greenhouse–Geisser correction for within-subject factors. An adjusted *p*-value of *p* < 0.05 was considered significant.

*TNFα* and *IL-6* were also increased in monocytes co-cultured with all three heat-killed postbiotics (LA:LR:SB 1:1:1) compared to monocytes cultured with both heat-killed bacteria (LA:LR 1:1) (Figure 4A,B, Tables S9 and S10). Only monocytes stimulated with heat-killed yeast (SB) exhibited considerable inter-personal variation in *IL-6* protein production. *IL-10* production showed high levels of inter-personal variation for monocytes cultured with postbiotics containing heat-killed bacteria, both individually and simultaneously administered (Figure 4C, Table S11). Of note, CD14^+^ monocytes co-cultured with heat-killed *L. rhamnosus* (LR) on average produced greater amounts of interleukin 1 alpha (*IL-1α*) and interleukin 1 beta (*IL-1β*) compared to any other postbiotic (Figure 4D,E, Tables S12 and S13). The competitive antagonist for IL-1 receptor, *IL-1RA*, was also increased in monocytes co-cultured with all three probiotic organisms (LA:LR:SB 1:1:1) compared to monocytes cultured with two heat-killed bacterial species (LA:LR 1:1) (Figure 4F, Table S14). However, individual co-culture with heat-killed *L. acidophilus* (LA) stimulated the greatest amount of *IL-1RA* production on average.

## 4. Discussion

Clinical interest in heat-killed preparations of various probiotic strains, including lactic acid bacteria and yeast, has grown due to their demonstrated efficacy in managing intestinal disorders and supporting therapies like *Helicobacter* infection and allergic respiratory diseases [24,25,26]. However, there is limited evidence regarding how heat-killed postbiotics, specifically in multi-strain formulations (e.g., *L. acidophilus 16axg*, *L. rhamnosus 18fx*, and *S. boulardii 16mxg)*, modulate immune responses or their underlying mechanisms of action. To address these gaps, our study investigates the immunomodulatory effects of heat-killed postbiotics in vitro on CD14^+^ monocytes to elucidate potential combinatorial attributes and contribute to understanding their potential mechanisms of action.

Previous studies investigating stimulation of naïve human monocytes reveal that these cells exhibit a biphasic immune response [27]. Upon encountering pathogens or heat-killed postbiotic stimuli containing MAMPs, monocytes initially mount a rapid proinflammatory response characterized by the production of cytokines including *TNFα*, *IL-1β*, and *IL-6*. Our data align with a biphasic immune response pattern, showing increased production of proinflammatory cytokines like *TNFα* approximately 2 h after exposure to heat-killed *L. acidophilus* and *L. rhamnosus* (Figure 1A,B). This immediate phase is critical for triggering the immune response and recruiting other immune cells to the site of infection or inflammation. We saw further evidence of this when examining the cytokines present in the supernatant after 24 h co-culture with the monocytes. Multiple CC chemokine ligands (*CCL4* and *CCL5*) and CXC chemokine ligands (*CXCL1*) were significantly elevated in monocytes treated with both heat-killed bacteria and yeast (LA:LR:SB 1:1:1), compared to monocytes given heat-killed bacteria without yeast (LA:LR 1:1) (Figure 3A,B). Monocytes interact with other immune cells through the release of cytokines, which are pivotal for regulating and coordinating immune responses [28]. As such, naïve monocytes are sensitive sentinels of immune modulation.

Following this initial proinflammatory phase, there is often a shift towards an anti-inflammatory response. This delayed phase is marked by increased production of anti-inflammatory cytokines such as *IL-10* and *TGF-β*, which aid in resolving inflammation, promoting tissue repair, and maintaining immune homeostasis [29]. Indeed, we observed a delayed onset and sustained elevation of the anti-inflammatory cytokine *IL-10*, peaking at 8 h, and remaining elevated compared to unstimulated monocytes for up to 24 h (Figure 1A,D). The magnitude of the delayed anti-inflammatory phase is proportional to the magnitude of the initial proinflammatory response. This demonstrates a potential regulatory feedback mechanism that ensures balanced immune responses to microbial stimuli, thereby preventing chronic inflammation and tissue damage. As we observed, the proinflammatory response returns to baseline levels shortly after the initial stimulation if no further stimulatory triggers are present, such as Damage-Associated Molecular Patterns (DAMPs) (Figure 1A). The duration and intensity of these immune response phases in vivo depend on various factors including the nature and dose of the stimulus, the specific immune cell types involved, and the immune tone of the tissue (e.g., activation vs. tolerance) [27].

The current study highlights that both heat-killed *L. acidophilus* and *L. rhamnosus* stimulate naïve monocytes without significant differences between them. However, *L. rhamnosus* tends to elicit a more consistent *IL-1α* and *IL-1β* cytokine response across different donors compared to *L. acidophilus* (Figure 4D,E). This aligns with previous findings suggesting that probiotics like *L. rhamnosus* can modulate cytokine production depending on the immune context [18]. For instance, they can enhance proinflammatory cytokine synthesis in the absence of inflammation while potentially suppressing it during excessive immune responses [30]. This also aligns with findings suggesting that strain-specific differences in immunomodulation as various postbiotics may possess distinct surface molecules, i.e., MAMPs that interact differently with PPRs on immune cells. These interactions can trigger specific signaling pathways leading to cytokine production. *L. rhamnosus* may have a more uniform pattern of MAMPs or a profile that consistently activates similar immune responses across different individuals [31,32].

We observed that the heat-killed yeast, *S. boulardii*, was notably less immunomodulatory compared with heat-killed bacteria at the same concentration (100 µg heat-killed cells/mL) (Figure 2D). This may be due to the saccharolytic effects of the heat-killing process, which can disrupt yeast cell surface MAMPs, such as β-glucans and lipomannans [33,34]. Interestingly, the addition of heat-killed yeast to heat-killed bacteria resulted in an enhancement of immune stimulation, possibly when combined with heat-killed postbiotics (Figure 2D). *S. boulardii* may potentiate their immunomodulatory effects by enhancing the recognition and response of immune cells to the probiotic components. Future research should explore these strain-specific effects and investigate potential additive effects when combining heat-killed postbiotics with *S boulardii*, aiming to enhance their overall immunomodulatory efficacy.

## 5. Conclusions

Postbiotics comprising single strains of *L. acidophilus*, *L. rhamnosus*, and *S. boulardii* bacteria were able to induce the production of both pro- and anti-inflammatory cytokines as well as chemokines in CD14^+^ monocytes. Both heat-killed *L. acidophilus* and *L. rhamnosus* stimulated naïve monocytes with few significant differences between them. Heat-killed *S. boulardii* stimulated less cytokine production compared to postbiotic bacteria at the same concentration. Interestingly, the addition of heat-killed yeast to heat-killed *L. acidophilus* and *L. rhamnosus* resulted in an enhancement of immune stimulation. Thus, heat-killed postbiotics have immune-modulating potential, particularly when bacteria and yeast are combined. Elucidating the immune-modulating properties of heat-killed postbiotics, particularly in combination cultures or fermented products, holds promise for developing targeted interventions that may fine-tune the immunomodulatory response and improve gut health. Continued research efforts should focus on refining these combinations and exploring their potential therapeutic applications to fully harness potential benefits for human health.

## Data Availability

The original contributions presented in the study are included in the article/Supplementary Materials; further inquiries can be directed to the corresponding author.

## Supplementary Materials

The following supporting information can be downloaded at https://www.mdpi.com/article/10.3390/life14121673/s1: Table S1: Primer and probe sequences for each of the four gene expression targets; Table S2: Thermocycler conditions used for RT-qPCR; Table S3: Adjusted *p*-values for *TNFα* gene expression by CD14^+^ monocytes 2 h after postbiotic stimulation; Table S4: Adjusted *p*-values for *IL-6* gene expression by CD14^+^ monocytes 4 h after postbiotic stimulation; Table S5: Adjusted *p*-values for *IL-10* gene expression by CD14^+^ monocytes 8 h after postbiotic stimulation; Figure S1: A heatmap showing the unscaled MFI values of each cytokine quantified via Luminex; Figure S2: Postzoning causes highly abundant cytokines to give a false negative signal; Figure S3: Individual donor variability is observed for several cytokines and chemokines; Table S6: Adjusted *p*-values for Mean Fluorescence Intensity measurements for *CCL4*, quantified by Luminex xMAP, produced by CD14^+^ monocytes 24 h after postbiotic stimulation; Table S7: Adjusted *p*-values for Mean Fluorescence Intensity measurements for *CCL5*, quantified by Luminex xMAP, produced by CD14^+^ monocytes 24 h after postbiotic stimulation; Table S8: Adjusted *p*-values for Mean Fluorescence Intensity measurements for *CXCL1*, quantified by Luminex xMAP, produced by CD14^+^ monocytes 24 h after postbiotic stimulation; Table S9: Adjusted *p*-values for Mean Fluorescence Intensity measurements for *TNFα*, quantified by Luminex xMAP, produced by CD14^+^ monocytes 24 h after postbiotic stimulation; Table S10: Adjusted *p*-values for Mean Fluorescence Intensity measurements for *IL-6*, quantified by Luminex xMAP, produced by CD14^+^ monocytes 24 h after postbiotic stimulation; Table S11: Adjusted *p*-values for Mean Fluorescence Intensity measurements for *IL-10*, quantified by Luminex xMAP, produced by CD14^+^ monocytes 24 h after postbiotic stimulation; Table S12: Adjusted *p*-values for Mean Fluorescence Intensity measurements for *IL-1α*, quantified by Luminex xMAP, produced by CD14^+^ monocytes 24 h after postbiotic stimulation; Table S13: Adjusted *p*-values for Mean Fluorescence Intensity measurements for *IL-1β*, quantified by Luminex xMAP, produced by CD14^+^ monocytes 24 h after postbiotic stimulation; Table S14: Adjusted *p*-values for Mean Fluorescence Intensity measurements for *IL-1 receptor antagonist*, quantified by Luminex xMAP, produced by CD14^+^ monocytes 24 h after postbiotic stimulation.
