# Supplementary material for: Immunomodulatory Properties of Multi-Strain Postbiotics on Human CD14+ Monocytes
Source: Life (Basel). 2024 Dec 17;14(12):1673. doi: 10.3390/life14121673 (PMC11728152; doi:10.3390/life14121673)
Supplement: Supplementary file 1 [file life-14-01673-s001.zip › life-3333283-supplementary.pdf]

Supplementary

Table S1. Primer and probe sequences for each of the four gene expression targets.

Target	Probe Sequence	Probe Target Region	Forward Primer	Reverse Primer	Reporter Dye	Quencher
IL-10	CGTGGAGC AGGTGAAGA ATGCCTTTA	Exon 4-Exon 5 Junction	CGCTGTC ATCGATT TCTTC	GGCTTTG TAGATGC CTTT	FAM	3' Iowa Black FQ Int ZEN
TNFα	AAACAACCC TCAGACGCC ACATCC	Exon 1	GGGAGAG AAGCAAC TACAG	GTCAGTA TGTGAGA GGAAGAG	HEX	3' Iowa Black FQ Int ZEN
IL-6	AGAGTAGTG AGGAACAAG CCAGAGCT	Exon 4	TAGAGTA CCTCCAG AACAGA	GGAACTG GATCAGG ACTTT	Tex 615	3' Iowa Black RQ
GAPDH	CTGGCCAAG GTCATCCAT GACAACTT	Exon 7	CCTCAAG ATCATCA GCAATG	ATGAGTC CTTCCAC GATAC	Cy5.5	3' Iowa Black RQ

Table S2. Thermocycler conditions used for RT-qPCR.

Step	Scan (y/n)	˚C	m:s	Loops	Rate (˚C/s)
1	N	25	2:00	-	8
2	N	50	15:00	-	8
3	N	95	2:00	-	8
4	N	95	00:03	44	8
5	Y	55	00:30	44	6

Table S3. Adjusted P values for *TNFα* gene expression by CD14[+] monocytes 2 hours after postbiotic stimulation.

group1	group2	p	p.adj	p.adj.signif
SB	LA	0.0001770	0.002000	**
SB	LR	0.0005540	0.006000	**
SB	LA:LR 1:1	0.0020000	0.022000	*
SB	LA:LR:SB 1:1:1	0.0110000	0.053000	ns
SB	NEG	0.0060000	0.033000	*
LA	LR	0.7580000	0.758000	ns
LA	LA:LR 1:1	0.0040000	0.028000	*
LA	LA:LR:SB 1:1:1	0.0490000	0.194000	ns
LA	NEG	0.0001710	0.002000	**
LR	LA:LR 1:1	0.0030000	0.028000	*
LR	LA:LR:SB 1:1:1	0.1620000	0.486000	ns
LR	NEG	0.0000989	0.001000	**
LA:LR 1:1	LA:LR:SB 1:1:1	0.3240000	0.648000	ns
LA:LR 1:1	NEG	0.0000489	0.000734	***
LA:LR:SB 1:1:1	NEG	0.0010000	0.012000	*

* : $0.01 \leq p < 0.05$

** : $0.001 \leq p < 0.01$

*** : $0.0001 \leq p < 0.001$

**** : $p < 0.0001$

Table S4. Adjusted P values for *IL-6* gene expression by CD14^{+} monocytes 4 hours after postbiotic stimulation.

group1	group2	p	p.adj	p.adj.signif
SB	LA	0.0000204	0.000306	***
SB	LR	0.0003600	0.004000	**
SB	LA:LR 1:1	0.0008780	0.007000	**
SB	LA:LR:SB 1:1:1	0.0040000	0.028000	*
SB	NEG	0.0180000	0.088000	ns
LA	LR	0.8070000	1.000000	ns
LA	LA:LR 1:1	0.0100000	0.057000	ns
LA	LA:LR:SB 1:1:1	0.4640000	1.000000	ns
LA	NEG	0.0002060	0.002000	**
LR	LA:LR 1:1	0.0003450	0.004000	**
LR	LA:LR:SB 1:1:1	0.4140000	1.000000	ns
LR	NEG	0.0000538	0.000753	***
LA:LR 1:1	LA:LR:SB 1:1:1	0.0940000	0.375000	ns
LA:LR 1:1	NEG	0.0001690	0.002000	**
LA:LR:SB 1:1:1	NEG	0.0007590	0.007000	**

* : $0.01 \leq p < 0.05$

** : $0.001 \leq p < 0.01$

*** : $0.0001 \leq p < 0.001$

**** : $p < 0.0001$

Table S5. Adjusted P values for *IL-10* gene expression by $CD14^+$ monocytes 8 hours after postbiotic stimulation.

group1	group2	p	p.adj	p.adj.signif
SB	LA	0.013000	0.101	ns
SB	LR	0.004000	0.038	*
SB	LA:LR 1:1	0.006000	0.057	ns
SB	LA:LR:SB 1:1:1	0.017000	0.121	ns
SB	NEG	0.029000	0.144	ns
LA	LR	0.019000	0.121	ns
LA	LA:LR 1:1	0.402000	0.402	ns
LA	LA:LR:SB 1:1:1	0.146000	0.394	ns
LA	NEG	0.000674	0.009	**
LR	LA:LR 1:1	0.004000	0.038	*
LR	LA:LR:SB 1:1:1	0.103000	0.394	ns
LR	NEG	0.000226	0.003	**
LA:LR 1:1	LA:LR:SB 1:1:1	0.098000	0.394	ns
LA:LR 1:1	NEG	0.000391	0.005	**
LA:LR:SB 1:1:1	NEG	0.002000	0.020	*

* : $0.01 \leq p < 0.05$

** : $0.001 \leq p < 0.01$

*** : $0.0001 \leq p < 0.001$

**** : $p < 0.0001$

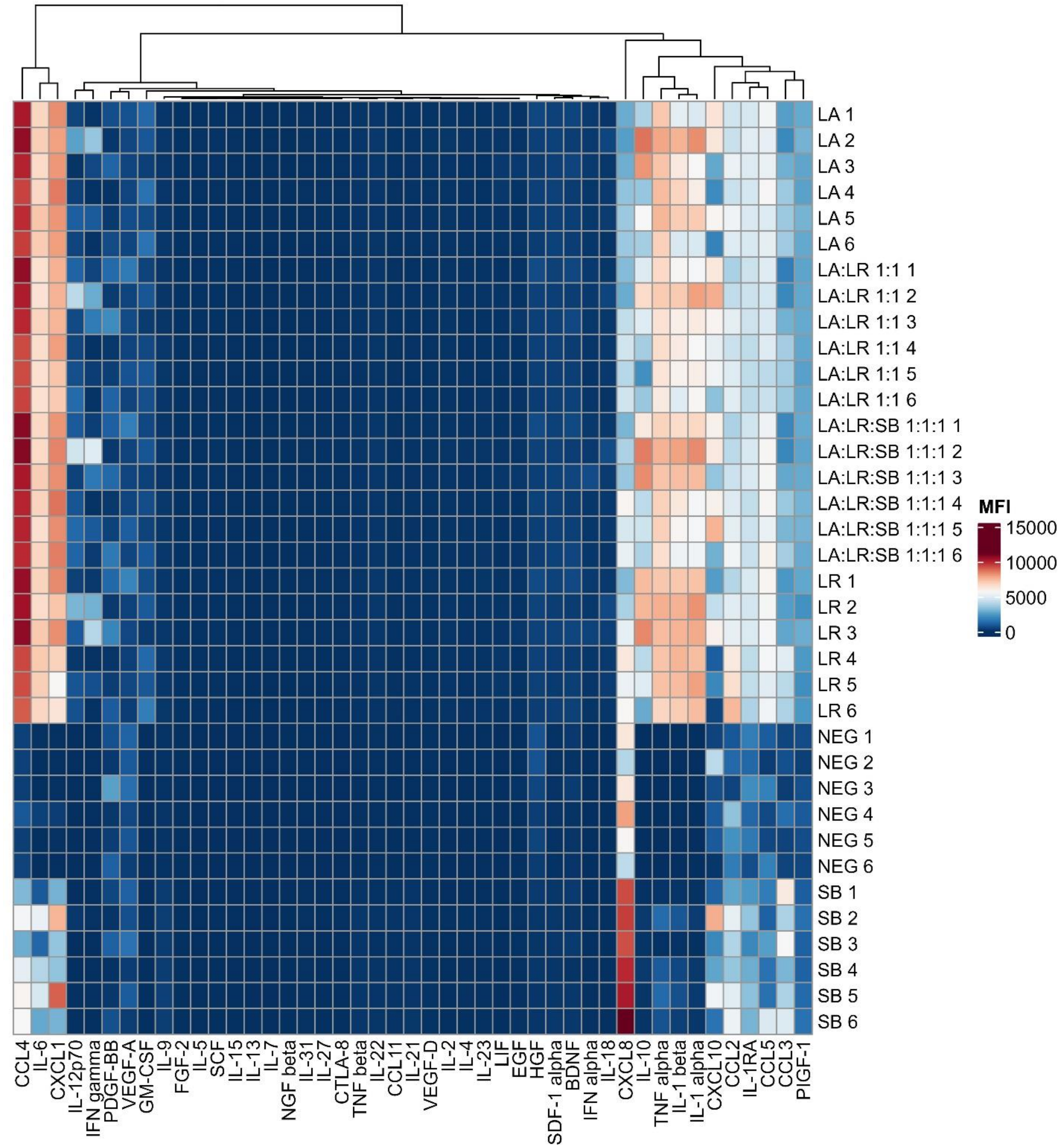

Figure S1. A heatmap showing the unscaled MFI values of each cytokine quantified via Luminex.

Squares are colored according to the raw MFI measured for each cytokine in each sample.

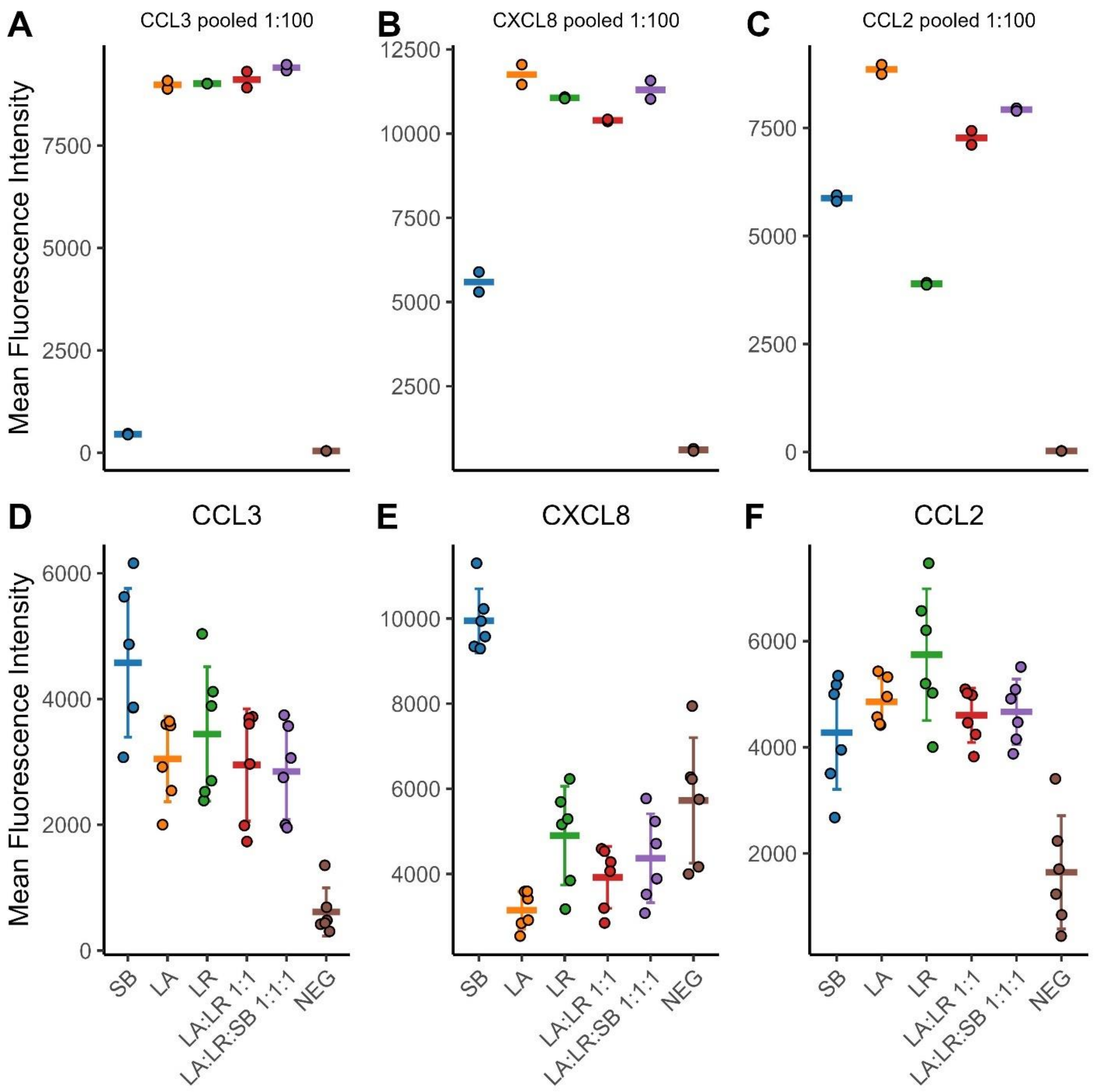

Figure S2. Postzoning causes highly abundant cytokines to give a false negative signal.

Highly abundant cytokines may form complexes that limit the accessibility of the antigen binding site targeted by the antigen-specific antibody in the Luminex panel. Each dot in Figure S2A-C represents the fluorescent intensity from each sample containing equal volumes of supernatant from each donor diluted 1:100 with fresh culture media. Each dot in Figure S2D-F represents the fluorescence intensity from individual donor's supernatant (n=6). The thick colored line indicates the sample mean for all donors, error bars represent the sample mean +/- standard deviation.

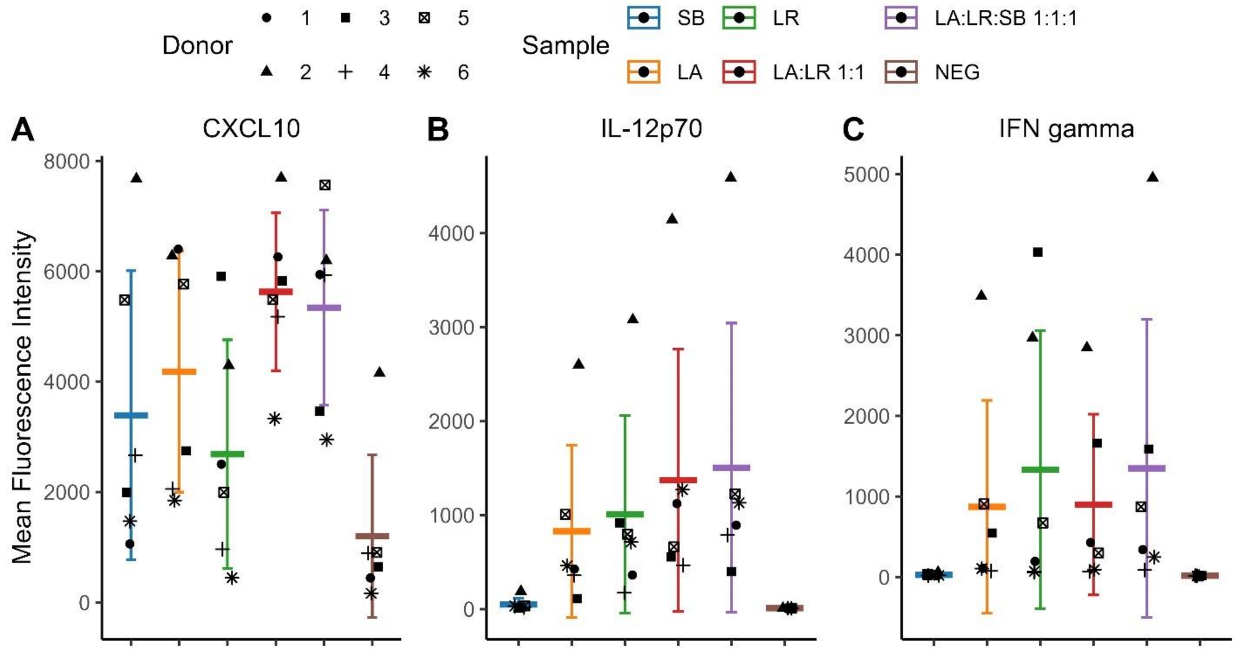

Figure S3. Individual donor variability is observed for several cytokines and chemokines.

Specifically, donor 2, represented by a black triangle, was a consistent outlier for several cytokines with anti-viral activity, namely *CXCL10*, *IL-12p70*, and *IFNγ*.

Table S6. Adjusted P values for Mean Fluorescence Intensity measurements for *CCL4*, quantified by Luminex xMAP, produced by CD14^{+} monocytes 24 hours after postbiotic stimulation.

group1	group2	p	p.adj	p.adj.signif
SB	LA	0.000331000	0.00300000	**
SB	LR	0.001000000	0.00800000	**
SB	LA:LR 1:1	0.000704000	0.00600000	**
SB	LA:LR:SB 1:1:1	0.000279000	0.00300000	**
SB	NEG	0.000485000	0.00400000	**
LA	LR	0.785000000	1.00000000	ns
LA	LA:LR 1:1	0.333000000	0.99900000	ns
LA	LA:LR:SB 1:1:1	0.004000000	0.02000000	*
LA	NEG	0.000000157	0.00000220	****
LR	LA:LR 1:1	0.610000000	1.00000000	ns
LR	LA:LR:SB 1:1:1	0.090000000	0.36000000	ns
LR	NEG	0.000001020	0.00001220	****
LA:LR 1:1	LA:LR:SB 1:1:1	0.003000000	0.01800000	*
LA:LR 1:1	NEG	0.000000292	0.00000380	****
LA:LR:SB 1:1:1	NEG	0.000000081	0.00000122	****

* : $0.01 \leq p < 0.05$

** : $0.001 \leq p < 0.01$

*** : $0.0001 \leq p < 0.001$

**** : $p < 0.0001$

Table S7. Adjusted P values for Mean Fluorescence Intensity measurements for *CCL5*, quantified by Luminex xMAP, produced by $CD14^+$ monocytes 24 hours after postbiotic stimulation.

group1	group2	p	p.adj	p.adj.signif
SB	LA	0.0040000	0.022000	*
SB	LR	0.0020000	0.015000	*
SB	LA:LR 1:1	0.0090000	0.047000	*
SB	LA:LR:SB 1:1:1	0.0010000	0.012000	*
SB	NEG	0.0240000	0.096000	ns
LA	LR	0.0280000	0.096000	ns
LA	LA:LR 1:1	0.0000776	0.000776	***
LA	LA:LR:SB 1:1:1	0.0680000	0.137000	ns
LA	NEG	0.0000359	0.000431	***
LR	LA:LR 1:1	0.0000132	0.000185	***
LR	LA:LR:SB 1:1:1	0.4520000	0.452000	ns
LR	NEG	0.0000155	0.000202	***
LA:LR 1:1	LA:LR:SB 1:1:1	0.0010000	0.012000	*
LA:LR 1:1	NEG	0.0000652	0.000717	***
LA:LR:SB 1:1:1	NEG	0.0000121	0.000182	***

* : $0.01 \le p < 0.05$

** : $0.001 \le p < 0.01$

*** : $0.0001 \le p < 0.001$

**** : $p < 0.0001$

Table S8. Adjusted P values for Mean Fluorescence Intensity measurements for *CXCL1*, quantified by Luminex xMAP, produced by CD14^{+} monocytes 24 hours after postbiotic stimulation.

group1	group2	p	p.adj	p.adj.signif
SB	LA	0.034000000000	0.23500000000	ns
SB	LR	0.165000000000	0.33000000000	ns
SB	LA:LR 1:1	0.070000000000	0.29400000000	ns
SB	LA:LR:SB 1:1:1	0.025000000000	0.19800000000	ns
SB	NEG	0.007000000000	0.06400000000	ns
LA	LR	0.059000000000	0.29400000000	ns
LA	LA:LR 1:1	0.000545000000	0.00600000000	**
LA	LA:LR:SB 1:1:1	0.083000000000	0.29400000000	ns
LA	NEG	0.000000000376	0.00000000534	****
LR	LA:LR 1:1	0.381000000000	0.38100000000	ns
LR	LA:LR:SB 1:1:1	0.044000000000	0.26200000000	ns
LR	NEG	0.000016000000	0.00019200000	***
LA:LR 1:1	LA:LR:SB 1:1:1	0.001000000000	0.01000000000	*
LA:LR 1:1	NEG	0.000000008160	0.00000010600	****
LA:LR:SB 1:1:1	NEG	0.000000000356	0.00000000534	****

* : $0.01 \le p < 0.05$

** : $0.001 \le p < 0.01$

*** : $0.0001 \le p < 0.001$

**** : $p < 0.0001$

Table S9. Adjusted P values for Mean Fluorescence Intensity measurements for *TNFα*, quantified by Luminex xMAP, produced by $CD14^+$ monocytes 24 hours after postbiotic stimulation.

group1	group2	p	p.adj	p.adj.signif
SB	LA	0.0000001680	0.000001850	****
SB	LR	0.0000007840	0.000007840	****
SB	LA:LR 1:1	0.0000018100	0.000014500	****
SB	LA:LR:SB 1:1:1	0.0000013000	0.000011700	****
SB	NEG	0.0210000000	0.042000000	*
LA	LR	0.1890000000	0.189000000	ns
LA	LA:LR 1:1	0.0003620000	0.002000000	**
LA	LA:LR:SB 1:1:1	0.0120000000	0.034000000	*
LA	NEG	0.0000000289	0.000000405	****
LR	LA:LR 1:1	0.0002960000	0.002000000	**
LR	LA:LR:SB 1:1:1	0.0050000000	0.019000000	*
LR	NEG	0.0000000158	0.000000237	****
LA:LR 1:1	LA:LR:SB 1:1:1	0.0040000000	0.018000000	*
LA:LR 1:1	NEG	0.0000000586	0.000000762	****
LA:LR:SB 1:1:1	NEG	0.0000000681	0.000000817	****

* : $0.01 \le p < 0.05$

** : $0.001 \le p < 0.01$

*** : $0.0001 \le p < 0.001$

**** : $p < 0.0001$

Table S10. Adjusted P values for Mean Fluorescence Intensity measurements for *IL-6*, quantified by Luminex xMAP, produced by CD14$^+$ monocytes 24 hours after postbiotic stimulation.

group1	group2	p	p.adj	p.adj.signif
SB	LA	0.00300000000	0.0270000000	*
SB	LR	0.00500000000	0.0370000000	*
SB	LA:LR 1:1	0.00700000000	0.0420000000	*
SB	LA:LR:SB 1:1:1	0.00600000000	0.0400000000	*
SB	NEG	0.00900000000	0.0460000000	*
LA	LR	0.98700000000	0.9870000000	ns
LA	LA:LR 1:1	0.04200000000	0.1680000000	ns
LA	LA:LR:SB 1:1:1	0.37000000000	0.7400000000	ns
LA	NEG	0.00000003220	0.0000003860	****
LR	LA:LR 1:1	0.00090400000	0.0100000000	**
LR	LA:LR:SB 1:1:1	0.10000000000	0.3000000000	ns
LR	NEG	0.00000000218	0.0000000327	****
LA:LR 1:1	LA:LR:SB 1:1:1	0.00100000000	0.0150000000	*
LA:LR 1:1	NEG	0.00000000431	0.0000000560	****
LA:LR:SB 1:1:1	NEG	0.00000000328	0.0000000459	****

* : $0.01 \leq p < 0.05$

** : $0.001 \leq p < 0.01$

*** : $0.0001 \leq p < 0.001$

**** : $p < 0.0001$

Table S11. Adjusted P values for Mean Fluorescence Intensity measurements for *IL-10*, quantified by Luminex xMAP, produced by CD14$^+$ monocytes 24 hours after postbiotic stimulation.

group1	group2	p	p.adj	p.adj.signif
SB	LA	0.002000	0.018	*
SB	LR	0.002000	0.015	*
SB	LA:LR 1:1	0.001000	0.014	*
SB	LA:LR:SB 1:1:1	0.001000	0.014	*
SB	NEG	0.005000	0.034	*
LA	LR	0.725000	1.000	ns
LA	LA:LR 1:1	0.163000	0.652	ns
LA	LA:LR:SB 1:1:1	0.495000	1.000	ns
LA	NEG	0.002000	0.018	*
LR	LA:LR 1:1	0.066000	0.330	ns
LR	LA:LR:SB 1:1:1	0.831000	1.000	ns
LR	NEG	0.001000	0.015	*
LA:LR 1:1	LA:LR:SB 1:1:1	0.018000	0.107	ns
LA:LR 1:1	NEG	0.001000	0.014	*
LA:LR:SB 1:1:1	NEG	0.000948	0.014	*

* : $0.01 \leq p < 0.05$

** : $0.001 \leq p < 0.01$

*** : $0.0001 \leq p < 0.001$

**** : $p < 0.0001$

Table S12. Adjusted P values for Mean Fluorescence Intensity measurements for *IL-1α*, quantified by Luminex xMAP, produced by CD14⁺ monocytes 24 hours after postbiotic stimulation.

group1	group2	p	p.adj	p.adj.signif
SB	LA	0.0000985000	0.000886000	***
SB	LR	0.0000000319	0.000000479	****
SB	LA:LR 1:1	0.0000109000	0.000142000	***
SB	LA:LR:SB 1:1:1	0.0000491000	0.000491000	***
SB	NEG	0.0180000000	0.091000000	ns
LA	LR	0.0150000000	0.091000000	ns
LA	LA:LR 1:1	0.8090000000	0.993000000	ns
LA	LA:LR:SB 1:1:1	0.4850000000	0.993000000	ns
LA	NEG	0.0001160000	0.000928000	***
LR	LA:LR 1:1	0.0020000000	0.013000000	*
LR	LA:LR:SB 1:1:1	0.0400000000	0.162000000	ns
LR	NEG	0.0000000596	0.000000834	****
LA:LR 1:1	LA:LR:SB 1:1:1	0.3310000000	0.993000000	ns
LA:LR 1:1	NEG	0.0000131000	0.000157000	***
LA:LR:SB 1:1:1	NEG	0.0000424000	0.000466000	***

* : $0.01 \leq p < 0.05$

** : $0.001 \leq p < 0.01$

*** : $0.0001 \leq p < 0.001$

**** : $p < 0.0001$

Table S13. Adjusted P values for Mean Fluorescence Intensity measurements for *IL-1β*, quantified by Luminex xMAP, produced by CD14$^+$ monocytes 24 hours after postbiotic stimulation.

group1	group2	p	p.adj	p.adj.signif
SB	LA	0.00001900000	0.0001900000	***
SB	LR	0.00000003310	0.0000004630	****
SB	LA:LR 1:1	0.00000246000	0.0000320000	****
SB	LA:LR:SB 1:1:1	0.00002240000	0.0002020000	***
SB	NEG	0.02200000000	0.1330000000	ns
LA	LR	0.03000000000	0.1500000000	ns
LA	LA:LR 1:1	0.31200000000	0.6240000000	ns
LA	LA:LR:SB 1:1:1	0.53300000000	0.6240000000	ns
LA	NEG	0.00003800000	0.0003040000	***
LR	LA:LR 1:1	0.00100000000	0.0070000000	**
LR	LA:LR:SB 1:1:1	0.05300000000	0.1880000000	ns
LR	NEG	0.00000000615	0.0000000922	****
LA:LR 1:1	LA:LR:SB 1:1:1	0.04700000000	0.1880000000	ns
LA:LR 1:1	NEG	0.00000434000	0.0000521000	****
LA:LR:SB 1:1:1	NEG	0.00001440000	0.0001580000	***

* : $0.01 \le p < 0.05$

** : $0.001 \le p < 0.01$

*** : $0.0001 \le p < 0.001$

**** : $p < 0.0001$

Table S14. Adjusted P values for Mean Fluorescence Intensity measurements for *IL-1 receptor antagonist*, quantified by Luminex xMAP, produced by $CD14^+$ monocytes 24 hours after postbiotic stimulation.

group1	group2	p	p.adj	p.adj.signif
SB	LA	0.00036000	0.0040000	**
SB	LR	0.00500000	0.0270000	*
SB	LA:LR 1:1	0.00300000	0.0230000	*
SB	LA:LR:SB 1:1:1	0.00200000	0.0140000	*
SB	NEG	0.01600000	0.0630000	ns
LA	LR	0.03800000	0.1150000	ns
LA	LA:LR 1:1	0.00055800	0.0060000	**
LA	LA:LR:SB 1:1:1	0.00400000	0.0250000	*
LA	NEG	0.00001350	0.0001650	***
LR	LA:LR 1:1	0.28400000	0.5680000	ns
LR	LA:LR:SB 1:1:1	0.29300000	0.5680000	ns
LR	NEG	0.00000202	0.0000303	****
LA:LR 1:1	LA:LR:SB 1:1:1	0.00400000	0.0250000	*
LA:LR 1:1	NEG	0.00001270	0.0001650	***
LA:LR:SB 1:1:1	NEG	0.00000956	0.0001340	***

* : $0.01 \leq p < 0.05$

** : $0.001 \leq p < 0.01$

*** : $0.0001 \leq p < 0.001$

**** : $p < 0.0001$
